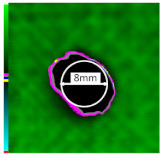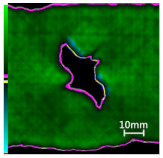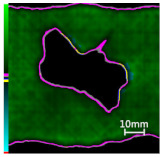# Investigating the Effect of Low-Temperature Drilling Process on the Mechanical Behavior of CFRP

**DOI:** 10.3390/polym14051034

**Published:** 2022-03-04

**Authors:** Hongxiao Wang, Xiaohui Zhang, Yugang Duan

**Affiliations:** 1 School of Mechanical and Electrical Engineering, Henan University of Technology, No.100 Lianhua Road, Gaoxin District, Zhengzhou 450001, China; zzwhx2019@haut.edu.cn; 2 State Key Lab for Manufacturing Systems Engineering, School of Mechanical Engineering, Xi’an Jiaotong University, No.99 Yanxiang Road, Xi’an 710054, China; ygduan@xjtu.edu.cn; 3 Henan Weihua Heavy Machinery Co., Ltd., No.18 Shanhai Road, Changyuan 453400, China

**Keywords:** lower temperature drilling process, mechanical behavior of CFRP, damage accumulation, static tensile tests, fatigue cyclic tests

## Abstract

Previous research has found that lower temperature drilling is helpful to improve the hole quality of carbon fiber reinforced polymer (CFRP). However, the influence of the lower temperature drilling process on the mechanical behavior of composites is yet not fully understood. To examine the influence of the lower temperature drilling process on the mechanical behavior of CFRP, the open hole CFRP specimens used for mechanical tests were obtained with three cases: drilling with −25 °C/uncoated carbide drills/(1000 rpm, 0.02 mm/r), 23 °C/coated carbide drills/(4000 rpm, 0.03 mm/r), and 23 °C/uncoated carbide drills/(1000 rpm, 0.02 mm/r), respectively; corresponding, three groups of open-hole specimens are obtained: specimens drilling at low-temperature with low damage, specimens drilling at room-temperature with low damage and specimens drilling at room-temperature with low damage; the mechanical behavior of the three groups specimens were obtained by static tensile, tensile–tensile fatigue cyclic tests and residual tensile strength test. The results have shown that the mechanical properties of specimens with a low-temperature drilling process is lower than those of the specimen with a normal drilling process due to the better drilling quality. The damage accumulation in specimens was increased with the damage degree of the original hole, the greater the damage degree, the worse the mechanical properties.

## 1. Introduction

Carbon fiber reinforced polymer (CFRP) materials are being widely used in aerospace, automotive and marine industries because of their high stiffness-to-weight and strength-to-weight ratios [1], strong design ability [2], good fatigue [3], and corrosion performance [4,5]. Although additive manufacturing is usually used to produce carbon fiber composite parts in the industry, its link parts need to be machined [6]. Drilling is a frequent machining process in the industry due to the need for component assembly [7]. However, due to the poor thermal conductivity of CFRP, the drilling area’s temperature is always far higher than the glass transition temperature of the resin matrix [8,9,10], causing drilling delamination, splitting, burr and other damage. According to refs. [11], when drilling area temperatures are higher than the glass transition temperature of the resin matrix, the interfacial shear strength of CFRP sharply decreases leading to large defects in the drilled hole surface. Meanwhile, the delamination is easily produced with the matrix degradation under the high drilling temperature.

To improve the drilling quality, cryogenic machining is used for machining CFRP and has made certain achievements in recent years [12,13,14]. For example, Bhattacharyya et al. [15] investigated the drilling process of composites under cryogenic conditions and reported that drilling quality was improved by cryogenic cooling. Xia et al. [16] investigated the effects of cryogenic cooling on drilling performance and surface integrity characteristics of CFRP and found that cryogenic cooling can enhance the surface integrity characteristics, but a high delamination factor when drilling under cryogenic conditions. Basmaci et el. [17] studied dipped cryogenic machining in drilling CFRP, the results indicated that the drilling of CFRP with the dipped cryogenic machining approach greatly improved machineability by reducing the surface roughness of the drilled parts and tool wear. However, it increased the thrust force and delamination factor. Nelson et al. [18] investigated cryogenic CO_2_ cooling in machining CFRP and CFRP/metal stack, and encouraging machining quality and efficiency were obtained. Khanna et al. [19] presented the results of cryogenic drilling of CFRP composites focusing on thrust force hole quality and inverse delamination factor, the results show Ra values of holes are considerably decreased by 14–38%, and the entry inverse delamination factor increased by 5–68% under cryogenic drilling when compared with dry drilling. Moreover, they advocate the suitability of cryogenic drilling for the industry. Based on the above opposite research conclusions, Jia et al. [20] studied the effects of the low-temperature method on milling CFRP in detail. In this study, the milling ambient temperature was segmented and proved that the cutting force and surface roughness remains relatively small between −10 °C and −25 °C. In our previous research [11], the relationship between ambient temperature, drilling zone temperature, and CFRP mechanical properties with temperature is studied in detail. The results show that the specimens with high hole wall quality and small delamination damage can be obtained at an appropriate drilling temperature range.

The above literature analysis shows that scholars have reached a consensus in the following five aspects, (1) high-temperature environment is not suitable for CFRP drilling; (2) Low-temperature technology is helpful to improving the drilling quality of hole wall; (3) The drilling axial force increases with the decrease of machining temperature; (4) too low a temperature will increase the drilling axial force and cause delamination damage; (5) machining damage was minimized when the drilling of CFRPs within the appropriate temperature range. So, the cryogenic cooling drilling method is recommended for machine CFRP [21]. However, the question of whether the variable temperature process under cryogenic drilling will affect the strength and fatigue life of CFRP is not considered.

Previous work has shown that residual stress always occurs when there is exposure to cryogenic temperatures or elevated temperatures [22] due to the difference of thermal expansion coefficient between fiber and resin matrix [23], and their heterogeneity on a microscopic and macroscopic scale [24]. The damage of many structures or mechanical parts is not only caused by external loading, but also due to the existence of inappropriate residual stress, and the failure and fracture behavior caused by residual is a complex phenomenon at present [25]. When drilling CFRP at low temperatures, the temperature in the drilling area is alternating, which poses a potential risk in the CFRP composites servicing. So, it is necessary to investigate the effect of the low-temperature drilling process on the mechanical behavior of CFRP.

This study describes in detail the cryogenic drilling tests of CFRP composites to investigate the influence of the low-temperature drilling process on the mechanical behavior of CFRP composites. By applying the cryogenic gas, the drilling ambient temperatures were controlled at −25 °C and room temperature, and get the specimens under specific process parameters. The specimens with the same drilling damage degree at different drilling temperatures were obtained to study the effect of the low-temperature drilling process on the mechanical properties of CFRP; the specimens with the different drilling damage degrees at the same drilling temperatures were obtained to study the effect of the damage degree on the mechanical properties of CFRP; Static and fatigue tests were performed to analyze damage evolution, damage accumulation.

## 2. Material and Methods

### 2.1. Specimen Details

**Figure 1:**
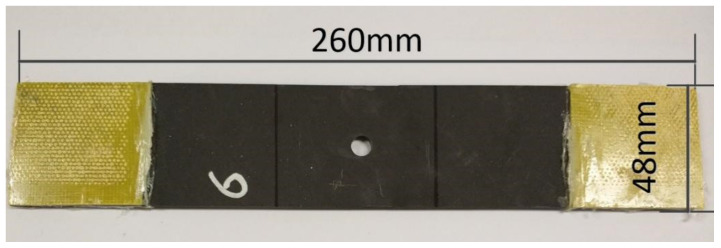
Size of CFRP open hole specimens with tensile and compress case.

According to the ASTM D5766 “Standard Test Method for open-hole Tensile Properties of polymer matrix composite laminates” the dimensions of the specimens are 300 mm long, 48 mm wide, and 3 mm thick (Figure 1). Five specimens were made for each temperature of the test. The same specimen stacking sequence and dimensions were used for both static and fatigue tests.

The composites specimens were made of T300/epoxy tape prepregs with a stacking sequence [45/0/−45/90]_3s_, and the single tape prepreg thickness is 0.125 mm. The tape prepregs were obtained from Hengshen CO. Ltd., Zhenjiang, China. The mechanical properties of carbon/epoxy composite at room temperature are given in Table 1. All holes were situated at the center of the plates.

**Figure 2:**
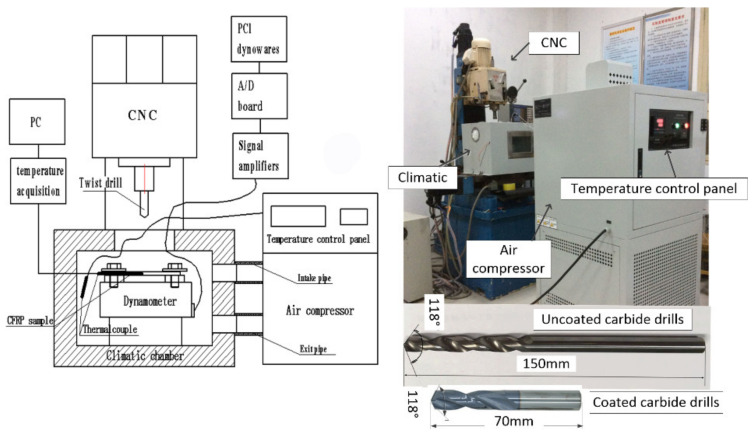
Schematic of temperature-controlled drilling experiment setup.

The drilling temperatures were controlled at −25 °C and room temperature at 23 °C. Temperature-controlled drilling tests were carried out by a cooling drilling system (Figure 2), which was composed of a vertical drilling-milling machine, climatic chamber, cryogenic cooling devices, and temperature measuring devices. Several uncoated and coated carbide conventional twist drills were used in this study, and the diameter is 8 mm, the point angle of this drill is 118°.

To study the effect of the low-temperature drilling process on the mechanical properties of CFRP, two groups of specimens with the same damage factor need to be obtained at low temperature and room temperature, respectively. To study the effect of damage degree on the mechanical properties of CFRP, two groups of specimens with different damage factors need to be obtained at the same ambient temperature. According to drilling experience, three sets of specimens under different drilling temperatures with different drilling process parameters and drills were made. In the case of low-temperature low damage (short for LTLD): set the spindle speed to 1000 rpm, set the feed rate to 0.02 mm/r and drilling holes under −25 °C with uncoated carbide drills. In the case of room temperature low damage (short for RTLD): set the spindle speed to 4000 rpm, set the feed rate to 0.03 mm/r and drilling holes under 23 °C with coated carbide drills. In the case of room temperature high damage (short for RTHD): set the spindle speed to 1000 rpm, set the feed rate to 0.02 mm/r, and drill holes under 23 °C with uncoated carbide drills. Detailed processing conditions and corresponding damage are shown in Table 2.

### 2.2. Drilling Quality Measure

To investigate the drilling surface quality of machined CFRP under different cases, the laser scanning confocal microscope (OLS400) was used in this study to measure the average surface roughness (Ra), respectively feed direction and vertical feed direction. To study the relationship of delamination areas versus drilling zone temperature; the scanning acoustic microscope (SAM D9500) was used in this study to measure the delamination areas.

### 2.3. Mechanical Testing

**Figure 3:**
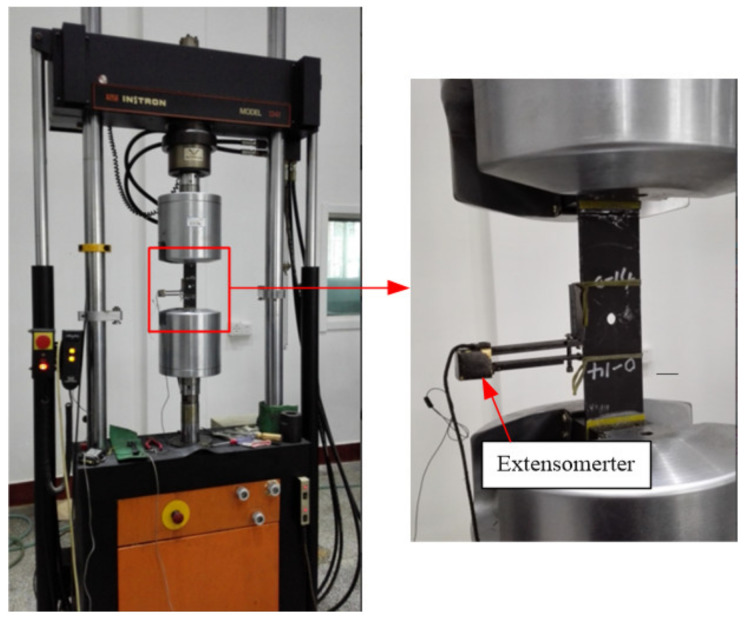
The universal testing machine for static and fatigue tests.

All the static tensile tests, tensile–tensile fatigue tests were performed on a universal testing machine (INSTRON model 1341) with a 100KN load cell (Figure 3). According to the ASTM D5766, the crosshead speed of the testing machine was 2 mm/min for static tensile and compress tests. An extensometer was used to measure the stress–strain curve of static tensile, and five specimens were performed for each static case. The test results were used to determine the ultimate tensile strength (UTS). Axial tensile–tensile fatigue tests were performed with a maximum stress level of 70% of UTS, a loading frequency of 10 Hz, and a stress ratio of 0.1, and three specimens were performed.

## 3. Results and Discussion

### 3.1. Drilling Quality under Various Drilling Cases

#### 3.1.1. Drilling Delamination Damage

**Figure 4:**
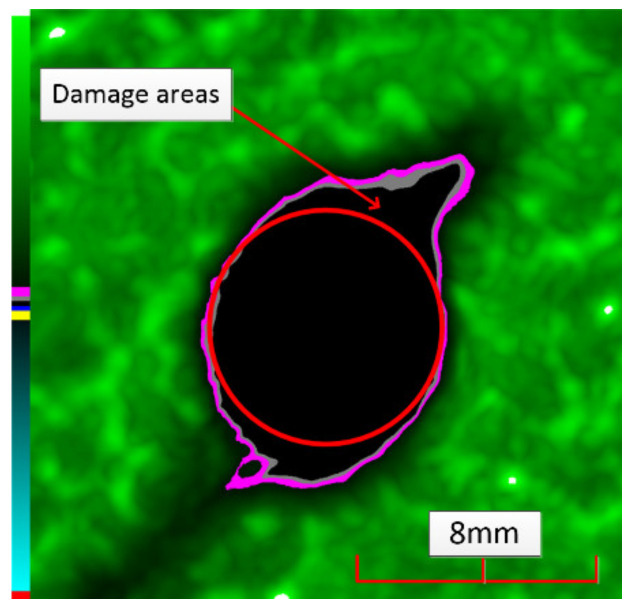
Ultrasonic scanning image of CFRP drilling delamination.

Delamination is the most serious damage during the service process of CFRP. Previous studies have indicated delamination damage has the greatest effect on the mechanical properties of composites among all the drilling damage [16,17]. An appropriate low-temperature drilling process can reduce the delamination damage [11,20], to compare the influence of a low-temperature drilling environment on the mechanical properties of CFRP, there must be a group of specimens drilling at room temperature, and the delamination damage degree is similar to it. Therefore, the delamination damage factor of the original hole used in the mechanical experiment must be evaluated. The ultrasonic scanner is used to measure the delamination inside the CFRP hole. The typical ultrasonic scanning measurement picture is shown in Figure 4. The red round hole in the figure is the drilling hole in the ideal state, and the other black parts are delamination damaged. The delamination factor (*f_d_*) is used to characterize the delamination damage process at the outlet. The calculation formula is as follows:

(1) $$f_{d}=\frac{S_{d}}{S}$$

where *S_d_* is the area caused by delamination damage and *S* is the ideal area of the hole.

Table 3 shows the images of CFRP drilling delamination damage by ultrasonic scanning under three cases. It is observed from Table 3 that the case LTLD when the process parameter is 1000 rpm, uncoated cemented carbide bit and drilling in −25 °C, the delamination damage of CFRP drilling is small, according to Formula 2, the delamination damage is about 6.4–12.5%; when the process parameter is 4000 rpm, 0.03 mm/r, with coated cemented carbide bit and drilling in 23 °C, the case RTLD delamination damage degree of CFRP drilling is similar to case LTLD, about 7.1–12.7%. The above two groups of results provide a basis for the comparison of the effects of drilling ambient temperature on static mechanical properties under the same damage degree. When the process parameter is 1000 rpm, 0.02 mm/r, with uncoated cemented carbide bit and drilling in 23 °C, the case RTHD delamination damage degree of CFRP drilling is higher than case LTLD, about 18.5–32%, it provides a basis for the comparison of the effects of damage degree on static mechanical properties under the same drilling ambient temperature.

#### 3.1.2. Drilling Surface Roughness

**Figure 5:**
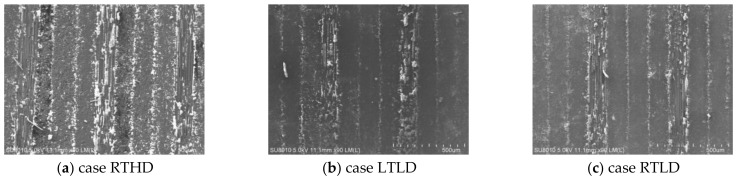
Micromorphology of surface quality of machined hole wall under different drilling cases.

**Figure 6:**
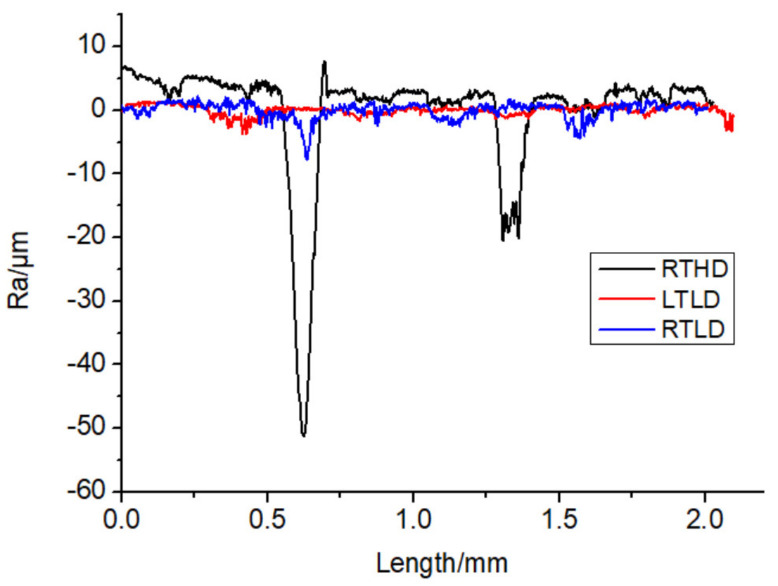
Curves of Ra along drilling direction under different drilling cases.

Some studies have indicated surface roughness of the hole wall has an effect on the fatigue mechanical properties of CFRP [23,26], the low-temperature drilling process can greatly improve the surface quality of the hole wall [12,13,14,15,16,17,18,19,20], to compare the influence of a low-temperature drilling environment on the mechanical properties of CFRP, there must be a group of specimens drilling at room temperature whose roughness is similar to the specimens drilling at low temperature. So, it is necessary to measure the roughness of the hole wall. Figure 5 illustrates the micromorphology of the hole wall under different drilling cases. Figure 6 shows the curves of Ra along the drilling direction under different drilling cases. It is observed from Figure 5 and Figure 6 that the surface quality of case RTHD is worst; due to the high rotating speed and better cutting tools, case RTLD has a similar high-quality surface quality of hole wall as that of low-temperature drilling case LTLD. It is providing a basis for the comparison of the effects of drilling ambient temperature on static mechanical properties under the same surface quality.

### 3.2. Results of Static Tensile and Tensile–Tensile Fatigue Analysis

**Figure 7:**
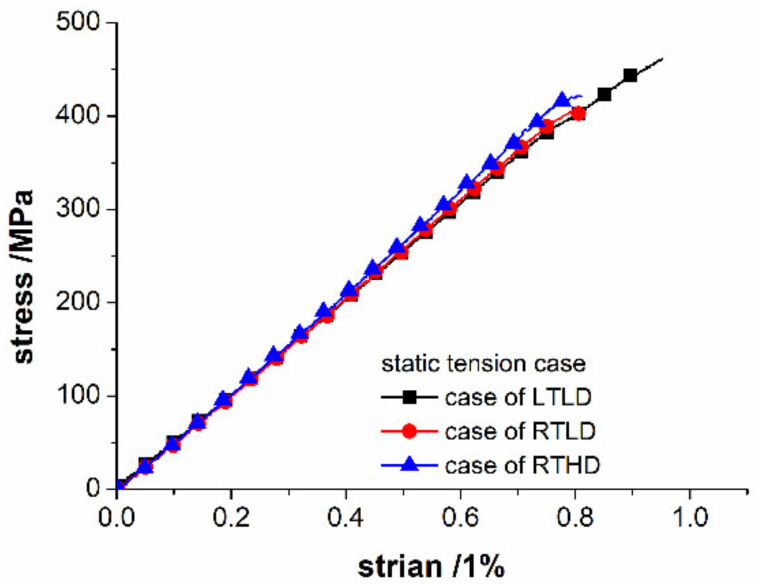
Representative curves of the stress versus strain for different cases of CFRP open hole static tensile.

**Figure 8:**
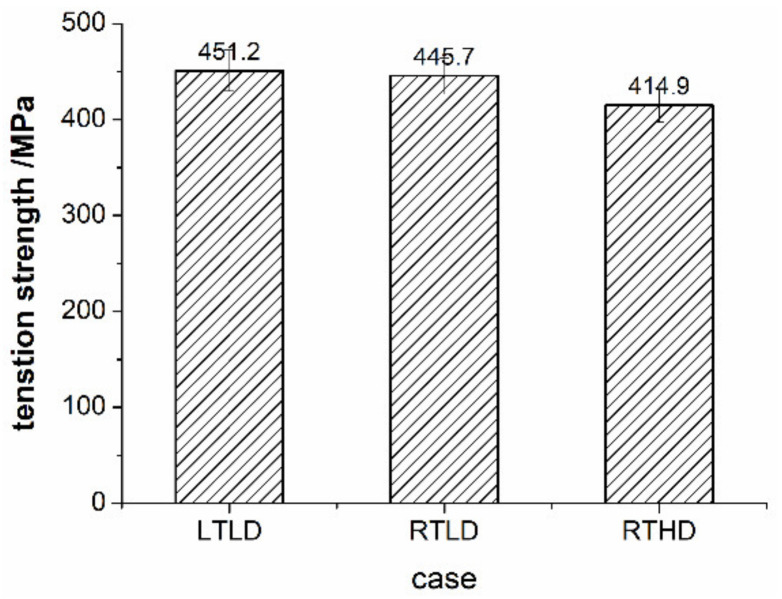
Static tensile strength of CFRP open hole under different cases.

Figure 7 shows the respective stress–strain curves for three series specimens under static loading. Due to the low toughness of CFRP, there is no obvious plastic deformation in the curve, and the slope of the curve is the tensile modulus of the material. It is observed from Figure 7, the ambient temperature and damage degree within this range have no significant effect on the tensile modulus of CFRP open-hole specimen, the elastic modulus of all cases is nearly, about 52,600 MPa. Figure 8 shows the tensile strength under different cases. The average failure stress under the case of RTLD is 445.7 MPa, LTLD is 451.2 MPa, RTHD is 414.9 MPa. It can be seen from Figure 8, when the delamination damage degree of the specimen is high, corresponding, the average failure stress is the worst: the value of the case LTLD (drilling with low temperature) and the case RTLD (drilling with high spindle speed and coated carbide drills) are almost the same, due to the similar drilling delamination damage; the value of case RTHD (drilling with room temperature, low spindle speed and uncoated carbide drills) is the lowest, due to the worst drilling delamination damage. It can be explained from the above static tensile results that the drilling ambient temperature has little effect on the static tensile mechanical properties of open-hole CFRP, the damage degree of the original hole is still the main factor affecting the static tensile mechanical properties of open-hole CFRP.

The load used in the dynamic tensile–tensile fatigue test is the UTS corresponding to the maximum ultimate strength in a static tensile test. To unify the boundary conditions, the fatigue load of all working conditions is based on the UTS of case LTLD (about 65 KN). During the test, 70% of the UTS (about 45 KN) is set as the maximum fatigue load, the loading frequency is 10 Hz and the stress ratio is 0.1. The ultrasonic scanning method was used to scan the specimens every 1,000,000 times, and the delamination damage propagation state of the specimens was recorded. Because CFRP has excellent tensile fatigue properties, it takes a long time to break the specimen at this load level, the fatigue test was stopped after 2,000,000 fatigue cycles, and then, a residual tensile strength test of incomplete failure specimens was carried out to compare the mechanical properties of perforated parts under different working conditions. The experimental method is the same as the static tensile test.

The damage evolution of tensile–tensile fatigue under different hole drilling temperatures and damage is shown in Table 4. It can be seen from Table 4 that the fatigue damage of tensile–tensile open-hole parts mainly occurs at the hole edge and two sides of the specimen. When the fatigue load is loaded to 2,000,000 times, the original delamination damage area of case LTLD is 10.4%, lower than the case RTLD (11.1%) and lower than the case RTHD (28%), and the tensile–tensile fatigue delamination damaged area of case LTLD is lower than case RTHD and case RTHD, the case RTHD with a large original delamination damage area also has the largest fatigue delamination damaged area. So, in terms of the delamination damaged area, the initial damage degree has the greatest influence on the tensile fatigue damage of perforated parts, the delamination damage area of the original hole is inversely proportional to the fatigue delamination damage area. Low-temperature drilling technology can reduce the delamination area of the original hole, and then reduce the fatigue delamination damage.

**Figure 9:**
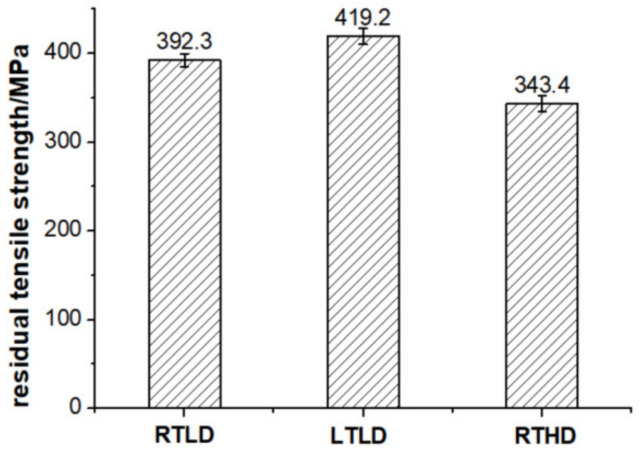
Residual tensile strength of CFRP open hole under different cases.

**Figure 10:**
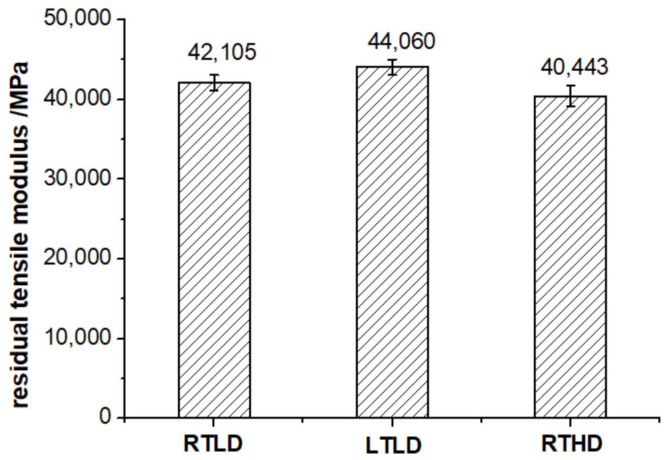
Residual tensile modulus of CFRP open hole under different cases.

Comparing the experimental results of residual tensile strength and modulus (Figure 9 and Figure 10), it is found that the mechanical properties of case LTLD are the best, case RTLD is the second, and RTHD is the worst. The residual tensile strength of case LTLD is 6.8% higher than that of case RTLD, and is 22.1% higher than that of case RTHD; the residual tensile modulus of case LTLD is 4.6% higher than that of case RTLD, and is 8.9% higher than that of case RTHD. The above results show that both ambient temperature and damage degree have effects on residual tensile strength and modulus, but damage degree has the greatest effect on tensile–tensile fatigue mechanical properties. When there is little difference in damage degree, the mechanical properties of case LTLD (low-temperature drilling specimens) is slightly better than case RTLD (drilling with room temperature, high spindle speed and coated carbide drills), the reason is that the overall drilling quality of the original hole drilled of case LTLD is slightly better than that of case RTLD: the delamination factor of case LTLD is slightly better than case RTLD (Table 3), and the Ra of case LTLD is slightly better than case RTLD (Figure 6). When the damage degree difference of the open-hole specimen is large, due to the better drilling quality of the low-temperature drilling process, the mechanical properties of low-temperature drilling low damage specimens are greater than room temperature drilling high damage specimens. So, the damage degree is the main factor affecting the tensile fatigue performance. The greater the damage degree is, the worse the tensile–tensile fatigue performance of the specimen is.

## 4. Conclusions

This study has experimentally analyzed the effects of the low-temperature drilling process on the mechanical behavior of CFRP. The static tensile and tensile–tensile fatigue mechanical properties of CFRP open-hole specimens with different damage degrees and different drilling ambient temperatures were compared and studied. The following conclusions can be drawn from this study.

The influence of the low-temperature drilling process on the static tensile mechanical properties of CFRP depends on the drilling quality (drilling delamination and surface roughness), and the low-temperature environment (−25 °C) itself has little influence on the mechanical properties of CFRP. When the damage degree of the CFRP open-hole specimens is similar, the difference in static tensile strength between low-temperature drilling and the normal drilling process is very small. When the damage degree of those specimens varies greatly, the static tensile strength of the CFRP open-hole specimens with a low-temperature drilling process is better than those of the specimens with a normal drilling process due to the better drilling quality.

The results of tensile–tensile fatigue testing have shown that the damage caused by the low-temperature drilling process is lower than those of the specimen with a normal drilling process due to the small drilling delamination damage. The damage accumulation in specimens was increased with the damage degree of the original hole. When there is little difference in damage degree of the CFRP open-hole specimens, the residual tensile strength and modulus of low-temperature drilling case are slightly higher than room-temperature drilling case, the reason is that the overall drilling quality of the original hole drilled at low temperature is slightly better than that drilled at room temperature. When the damage degree difference of the open-hole specimen is large, the residual tensile strength and modulus of low-temperature drilling case are obviously higher than room-temperature drilling case, the reason is that the overall drilling quality of the original hole drilled at low temperature is better than that drilled at room temperature.

So, the damage degree is the main factor affecting the tensile fatigue performance. The greater the damage degree, the worse the tensile fatigue performance of specimens. Drilling at a low temperature can improve the drilling quality of CFRP holes, it is an advantage to the static tensile and tensile–tensile fatigue mechanical behavior of CFRP open-hole specimens.

## Figures and Tables

**Table 1:** Mechanical properties of T300/epoxy composite at room temperature.

Elastic Properties	CFRP
Longitudinal modulus, $E_{11}$ (MPa)	137,000
Transverse modulus, $E_{22}$ (MPa)	9000
Poisson’s ratio, $\upsilon_{12}$	0.28
Shear modulus, $G_{12}$ (MPa)	6000
Fiber content (%)	60 ± 2

**Table 2:** Test cases of mechanical properties of CFRP open-hole parts.

Case	Drilling Ambient Temperature	Damage Condition	Drilling Process Parameters	Drills Material
Static tensile of LTLD	−25 °C	low	1000 rpm, 0.02 mm/r	uncoated carbide drills
Static tensile of RTLD	23 °C	Low	4000 rpm, 0.03 mm/r	Coated carbide drills
Static tensile of RTHD	23 °C	high	1000 rpm, 0.02 mm/r	uncoated carbide drills
Tensile–tensile fatigue of LTLD	−25 °C	low	1000 rpm, 0.02 mm/r	uncoated carbide drills
Tensile–tensile fatigue of RTLD	23 °C	Low	4000 rpm, 0.03 mm/r	Coated carbide drills
Tensile–tensile fatigue of RTHD	23 °C	high	1000 rpm, 0.02 mm/r	uncoated carbide drills

**Table 3:** Images of CFRP drilling delamination damage by ultrasonic scanning.

Cases	Ultrasonic Scanning Image of CFRP Drilling Delamination
LTLD −25 °C drilling; 1000 rpm, 0.02 mm/r; Uncoated carbide drills; Damage factor: 6.4–12.5%	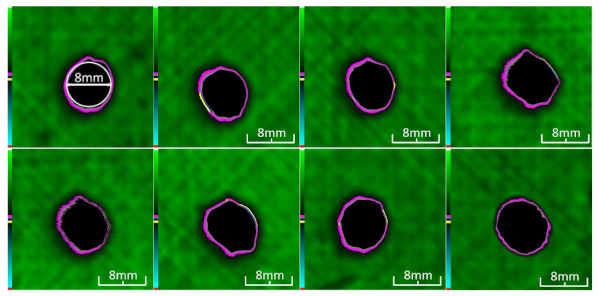
RTLD: 23 °C drilling; 4000 rpm, 0.03 mm/r; Coated carbide drills; Damage factor: 7.1–12.7%	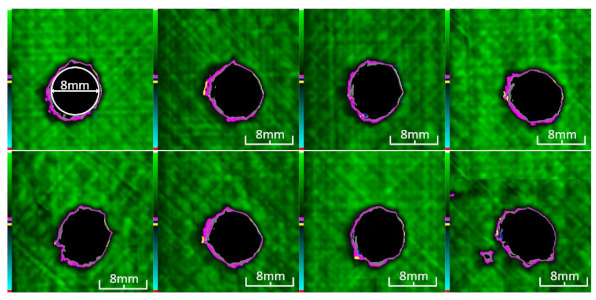
RTHD 23 °C drilling; 1000 rpm, 0.02 mm/r; Uncoated carbide drills; Damage factor:18.5–32%	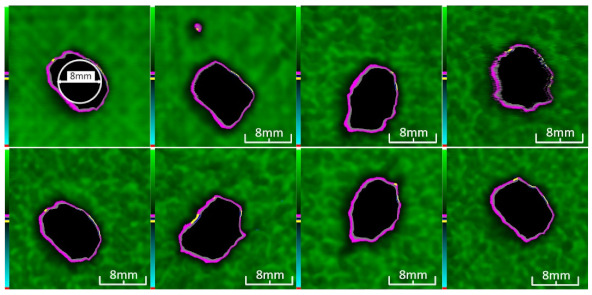

**Table 4:** C-scan image showing the tensile–tensile fatigue damage evaluation in the different drilling cases under 1,000,000 and 2,000,000 cycle loads.

Case	Original Hole	N = 1,000,000	N = 2,000,000
Tensile–tensile fatigue of LTLD; Original delamination damage factor: 10.4%; Maximum fatigue load: 45 Kn	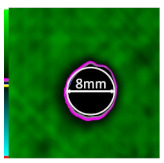	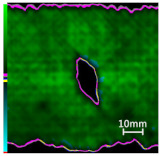	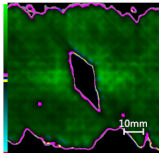
Tensile–tensile fatigue of RTLD; Original delamination damage factor: 11.1%; Maximum fatigue load: 45 Kn	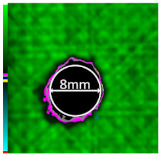	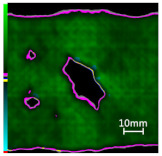	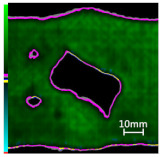
Tensile–tensile fatigue of RTHD; Original delamination damage factor: 28%; Maximum fatigue load: 45 Kn